# Tumour cell derived effects on monocyte/macrophage polarization and function and modulatory potential of *Viscum album* lipophilic extract in vitro

**DOI:** 10.1186/s12906-015-0650-3

**Published:** 2015-04-24

**Authors:** Myriam Estko, Stephan Baumgartner, Konrad Urech, Matthias Kunz, Ursula Regueiro, Peter Heusser, Ulrike Weissenstein

**Affiliations:** Witten/Herdecke University, 58448 Witten, Germany; Society for Cancer Research, Hiscia Institute, 4144 Arlesheim, Switzerland; Institute of Integrative Medicine, Witten/Herdecke University, 58448 Witten, Germany

**Keywords:** Macrophage polarization, Monocyte chemotactic transmigration, Tumour microenvironment, *Viscum album* lipophilic extract (VALE), Mistletoe

## Abstract

**Background:**

Macrophages are highly versatile cells that play an important role in tumour microenvironment. Tumour associated macrophages (TAMs) have been linked to both, good or bad prognosis of several cancer types depending on their number, composition and polarization. *Viscum album* lipophilic extract (VALE) contains several pentacyclic triterpenes known to modulate the activity of monocytes and other immune cells and to exhibit anticancer properties. In our *in vitro* study, we investigated the effect of tumour cell lines on macrophage polarization and monocyte chemotactic transmigration and examined the modulatory potential of VALE and its predominant triterpene oleanolic acid (OA).

**Methods:**

Human peripheral blood monocytes were differentiated into monocyte derived macrophages (MDM) using M-CSF and polarized into M1 by IFN-γ and LPS and into M2 macrophages by IL-4 and IL-13 or by co-culture with two different tumour cell lines. Polarized macrophages were subsequently treated with VALE or OA. Phenotypic markers and cytokines were assessed by flow cytometry and immunoanalysis. Migration of human peripheral blood monocytes induced by monocyte chemotactic protein-1 (MCP-1) or supernatants of different tumour cell lines under the influence of VALE or OA was measured in a chemotaxis transmigration assay.

**Results:**

*In vitro* polarized M1 and M2 type macrophages revealed specific phenotypic patterns and tumour cell co-cultured MDM displayed ambiguous phenotypes with M1 as well as M2 associated markers. VALE and OA showed modest influence on cell surface marker profile and cytokine expression of tumour cell co-cultured macrophages. All tumour cell supernatants markedly enhanced the migratory activity of monocytes. VALE and OA significantly inhibited MCP-1 induced monocyte transmigration, whereas monocyte migration initiated by tumour cell derived supernatants was not affected.

**Conclusions:**

In our study we reconfirmed that co-culture with different tumour cell lines can result in a mixed macrophage phenotype with M1 as well as M2 patterns, a finding that is important for a better understanding of tumour microenvironment functions. Moreover, we demonstrated that VALE shows slight immunomodulatory effects on tumour cell co-cultured macrophages and modulates monocyte chemotactic transmigration *in vitro*, indicating promising possibilities of triterpenes from *Viscum album* L. to contribute in a multimodal concept of anti-cancer therapy in future. Our data contribute to an understanding of monocyte function and macrophage polarization *in vitro* and of the possibility to influence their behaviour by triterpene containing mistletoe extracts.

## Background

The microenvironment of tumours is a subject of great interest in current investigations on cancer treatment and is known to play a crucial role in terms of neoangiogenesis, inflammation and modulation of the immune system [1-3]. It has been shown that macrophages play an important role in the microenvironment of tumours [4]. These heterogeneous cells are able to change their phenotype due to their environment [5], an effect that is especially seen within tumours [6,7]. Two major types are described at sites of inflammation: so-called classically activated pro-inflammatory M1 and alternatively activated immunosuppressive M2 macrophages. The M2 form of tumour infiltrating macrophages (TAMs) has been widely associated with poor prognosis, induction of angiogenesis, tissue remodelling and enhancement on metastasis growth and seeding [6,8,9]. However, *in vitro* studies with macrophages and tumour cells suggest a more complex situation, resulting in a functional polarization towards a mixed M1/M2 phenotype. [10-13]. Thus, investigation on how to modulate these cells alone or in presence of tumour cells is an important aspect in current research.

Natural products from plants became focus in search of new therapeutic options for combating tumours in a multimodal therapy concept [3,14,15]. Amongst these, pentacyclic triterpenes have shown effects on tumours not only by inducing tumour cell apoptosis [16], but also by modulating the immune system and showing anti-angiogenic and anti-inflammatory activities *in vitro* and *in vivo* [17-20].

Aqueous extracts of *Viscum album* L. (European white-berry mistletoe) are widely used in complementary cancer treatment [21], containing several bioactive compounds such as lectins, viscotoxins and polysaccharides [21]. Besides the hydrophilic active substances, the plant itself contains several pentacyclic triterpenes, among them oleanolic acid, betulinic acid, ursolic acid and lupeol [22,23]. Due to their insolubility in water these compounds are not represented in significant amounts in aqueous mistletoe extracts [24]. However, previous studies have shown apoptosis inducing and anti-angiogenic activity of these lipophilic mistletoe compounds *in vitro* and *in vivo* [20,22,25-28]. Furthermore, a lipophilic extract from *Viscum album* L. enhanced the release of pro-inflammatory cytokines and induced anti-apoptotic effects on human isolated peripheral blood monocytes *in vitro* [29].

The purpose of the present *in vitro* study was to investigate the effects of soluble tumour cell factors on polarization and chemotaxis/migration of monocytes and macrophages. Additionally the modulatory potential of VALE and its predominant triterpene oleanolic acid on these phenotypic and functional properties was evaluated.

## Methods

### Products investigated

Viscum album lipophilic extract (VALE), containing 9.3% oleanolic acid (OA) was kindly provided by Hiscia Institute, Verein für Krebsforschung, Arlesheim, Switzerland and obtained by ultra critical CO_2_ extraction. OA (purity > 98%) was purchased from Sigma-Aldrich (Buchs, Switzerland). Both reagents were dissolved in DMSO (Sigma-Aldrich, Buchs, Switzerland) prior to each experiment. Final concentrations of DMSO in all assays never exceeded the concentration of 1%.

Possible participation of contaminating mistletoe lectins and viscotoxins in VALE can be excluded, as both toxic proteins are insoluble in lipophilic solvents. In addition the heat labile mistletoe lectins [30] and viscotoxins [31] are expected to be inactivated during the extraction procedure of VALE (at least 5 h at 70°C).

### Isolation of peripheral blood monocytes

The study was approved by the Ethics Committee of Basel, Switzerland (EKBB). Blood samples were taken from 11 healthy donors after obtaining written informed consent. Venous blood was drawn into Li-Heparin tubes (Sarstedt, Sevelen, CH) and PBMC were isolated by standard ficoll-paque gradient centrifugation according to manufacturer´s protocol (GE Healthcare Bio-Sciences AB, Uppsala, Sweden). CD14 positive monocytes were obtained by MACS (Magnetic Activated Cell Sorting) separation technique (Miltenyi Biotec GmbH, Bergisch Gladbach, Germany) according to the manufacturer´s instructions. In brief, PBMC were magnetically labelled with CD14 microbeads, applied to a positive selection column and placed in the magnetic field of a QuadroMACS™ separation unit (Miltenyi Biotec GmbH, Bergisch Gladbach, Germany). After detachment from the cell separator, CD14+ cells were eluted and re-suspended in medium (RPMI 1640, supplemented with 10% FCS, 2 mM L-Glutamine and 1% Penicillin-Streptomycin, Sigma-Aldrich, Buchs, Switzerland). Purity and viability of the isolated cells were validated using FACS analysis prior to each assay. The purity of isolated CD14+ monocytes was >95% (data not shown).

### Cell culture and tumour cell conditioned medium (CM)

Human breast carcinoma cell lines MCF-7 and HCC1143, pancreas adenocarcinoma cell line PA-TU-8902, prostate >carcinoma cell line DU145 and lung carcinoma cell line NCI-H460 were obtained from DSMZ (German Collection of Microorganisms and Cell Cultures, Braunschweig, Germany).

In general RPMI 1640 medium, supplemented with 2 mM L-Glutamine and 1% Penicillin-Streptomycin and FCS (either 10% or 1% according to the assay) was used for monocyte/macrophage, NCI-H460, HCC1143 and DU145 cell culture. For MCF-7 cells we used Eagle´s MEM, supplemented with 10% FCS, 2 mM L-Glutamine, 1% Penicillin/Streptomycin and 1 mM Sodium Pyruvate and for PA-TU-8902 cells Dulbecco´s MEM High Glucose supplemented with 1 mM Sodium Pyruvate, 2 mM L-Glutamine, 1% Penicillin/Streptomycin and either 10% or 1% FCS was used (all reagents from Sigma, Buchs, Switzerland).

Cell lines were kept under standard culture conditions (37°C, 5% CO_2_ and 95% humidity), tumour cell conditioned media were obtained by 24 h incubation of 90% confluent tumour cells in 10 ml of fresh culture medium in 25 cm^2^ cell flasks. Cell free supernatants were carefully harvested, filtered through 0.5 μm filters and stored in aliquots at −80°C.

### Polarization of monocyte-derived macrophages (MDM) and treatment with VALE/OA

Monocytes were differentiated and polarized according to established protocols [32-34], with some modifications. All steps were performed using RPMI supplemented with 10% FCS. For differentiation of monocytes into macrophages, freshly isolated monocytes were seeded into wells of a 12 well plate (Corning) at a density of 0.6 – 1 × 10^6^/ 2 ml/well in RPMI medium supplemented with 25 ng/ml human macrophage colony stimulating factor (MCS-F) (Sigma-Aldrich, Buchs, Switzerland). The cells were incubated at 37°C, 5% CO_2_ for 10 days, after 3 days and 6 days, medium was partially replaced by fresh medium.

On day 10, differentiation medium was replaced by polarization media. 20 ng/ml IFN-γ and 100 ng/ml LPS were used to generate M1 and 20 ng/ml IL-4 and 20 ng/ml IL-13 to generate M2 polarized macrophages (all reagents from Sigma-Aldrich, Buchs, Switzerland). Cells treated with medium only were declared as M0. For the polarization of MDMs by tumour cells we used an indirect co-culture assay. 1 ml/well RPMI was added to the MDM containing wells. Transwell permeable inserts with a pore size of 0.4 μm (Corning, Tewksbury, MA, USA) were placed into the wells and tumour cells (NCI-H460 or MCF-7 respectively) were seeded at a density of 1.5 × 10^5^ cells/ml/insert. After 3 days of polarization the media of M2 and tumour cell polarized MDM were replaced by RPMI, either medium alone as control, or in combination with VALE 25 μg/ml or OA 2.5 μg/ml. M0 and M1 media were replaced by RPMI only. The cells were incubated for 3 more days before supernatants were collected and stored at −20°C until further analysis. Cells were trypsinized, washed and analysed by flow cytometry. Five independent experiments were evaluated.

### Flow cytometric analysis and cytokine detection

Measurement of cell surface marker expression (mean fluorescence intensity, MFI) and viability was performed by four-colour flow cytometry (FACS Calibur, BD Biosciences, San Jose, CA, USA) using specific antibodies for CD11b, CD14, CD36, CD206, CD40, HLA-DR and 7-AAD (BD Biosciences Pharmingen™, San Diego, CA, USA). Supernatants were analysed for TNF-α, IL-6 and IL-10 in an automated immunoanalyzer (Immulite 1000 Immunoassay System, Siemens, Schweiz).

### Transmigration/Chemotaxis assay

The effect of VALE and OA on transmigration/chemotaxis of human peripheral monocytes was investigated using FluoroBlok™ 96-Multiwell Insert Plates (Corning, Tewksbury, MA, USA). Freshly isolated monocytes were labelled with 1.5 μM Calcein AM (Corning, Tewksbury, MA, USA) in RPMI-medium (10% FCS) for 30 min at 37°C, 5% CO_2_. The fluorescent cells were washed and re-suspended in medium (1% FCS) at a density of 0.75 - 1,8 × 10^6^/ml. 50 μl of the cell suspension were added onto the polyethylene terephthalate (PET) membrane with 8 μm pores of FluoroBlock Multiwell inserts that was previously coated with 5 μg/well human fibronectin (hFN) (Corning, Tewksbury, MA, USA). Cells were allowed to rest for 15 min. Into a separate 96-square well flat bottom plate (Corning, Tewksbury, MA, USA), 200 μl/well of the reagents were added, either 25 nM MCP-1 alone as positive control or in combination with VALE 50 μg/ml or OA 5 μg/ml respectively, medium with 1% FCS was used as negative control and wells with medium without cells were used as blank. The multiwell inserts, containing the calcein labelled cells, were gently lowered into the reagent containing plate. Fluorescence was measured immediately and in 20 minutes intervals afterwards up to 5 hours. Measurements were performed with a bottom fluorescence plate reader (PerkinElmers Victor2 1420) at 485/530 nm (Ex/Em) wavelengths. Between measurements the plate was carefully stored at 37°C, 5% CO_2_. Four independent experiments performed in duplicates were evaluated. After subtraction of the blank, data were referred to the MCP-1-free negative control. For each replicate, a mean fluorescence value was calculated based on data obtained within 4 hours.

In a second step we examined the chemotactic ability of tumour cell conditioned media (CM) on monocytes. CMs of the tumour cell lines NCI-H460, HCC1143, DU-145 or PA-TU-8902 were thawed and 200 μl were applied to the respective well in duplicates. All tumour cell CMs were tested alone and in combination with VALE 50 μg/ml or OA 5 μg/ml respectively. The experimental steps were done as described above. Three to five independent experiments were performed for each cell line.

### Statistical analysis

All experimental data were evaluated by analysis of variance (ANOVA) with α = 0.05 and type VI decomposition. Pairwise comparisons were evaluated by the LSD-test only if the preceding global F-test was significant (protected Fisher’s LSD-test). This procedure yields a good security against false-positive as well as false-negative results [35]. All calculations were done with Statistica 6.0 (Statsoft, Tulsa, USA).

## Results

### Polarization of macrophages with tumour cell lines led to a mixed M1/M2 phenotype

**Figure 1:**
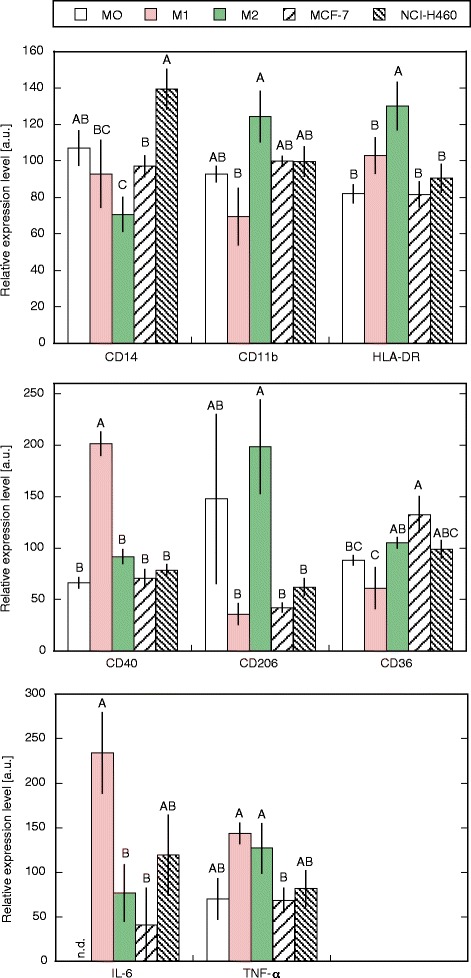
Cell surface marker and cytokine expression of polarized macrophages. CD14, CD11b, HLA-DR, CD40, CD206, CD36 cell surface expression and IL-6 and TNF-α release were measured by flow cytometry or immunoanalysis after M0, M1 or M2 polarization of differentiated macrophages or polarization by co-culture with MCF-7 or NCI-H460 tumour cell lines. Mean results of 5 independent experiments are shown. Data represent the relative MFI (arbitrary units, standardized to experimental mean = 100). Columns with distinct letters differ significantly (p < 0.05) from each other. n.d. = not detectable.

Freshly isolated monocytes were differentiated into MDM with M-CSF before polarization into M1 using IFN-γ and LPS or into M2 macrophages by IL-4 and IL-13, or were co-cultured with NCI-H460 or MCF-7 tumour cells to better mimic a realistic situation of macrophage polarization by tumours. MDM without polarization after M-CSF differentiation were declared as M0. The *in vitro* differentiation of human monocyte derived macrophages by M-CSF and the following polarization into M1- and M2-like cell types revealed specific phenotypic patterns. M1 macrophages significantly enhanced the relative expression of CD40 (p < 0.001) and IL-6 (p = 0.019) and M2 macrophages enhanced the relative expression of CD11b (p < 0.001), CD36 (p = 0.008), CD206 (p < 0.002) and HLA-DR (p = 0.028) compared to the opposite polarization phenotype. CD14 and TNF-α did not significantly differbetween M1 and M2 (Figure 1). For all polarization cell types, levels of IL-10 remained under detection limit in our experiments.

MDM co-cultured with NCI-H460 showed a significant increase in the expression of CD14 (p < 0.01) compared to M1, M2 and MCF-7 polarized macrophages. CD40 expression was significantly lower compared to M1 (p < 0.001), and expression of CD206 and HLA-DR was significantly lower compared to M2 (p < 0.01).

MCF-7 co-cultured macrophages exhibited significantly higher levels of CD36 (p < 0.001) and significantly lower levels of CD40 (p < 0.001) and IL-6 (p < 0.01) than M1. Expression of CD14 (p < 0.05) was significantly higher and CD206 (p < 0.01) and HLA-DR (p < 0.001) was significantly lower than in M2-like macrophages (Figure 1).

Our results confirm the particular *in vitro* polarization of macrophages into M1 or M2 phenotypes, displaying specific receptors and cytokine release. Notably, the expression of monocyte/macrophage markers CD11b and HLA-DR differed significantly between M1 and M2 polarized macrophages and TNF-α release was up regulated, although not significantly, in both, M1 and M2 compared to M0. Co-culture with tumour cell lines induced a mixed M1/M2 phenotype. Expression of CD36, CD40 and IL-6 was M2-like and levels of CD14, CD206 and HLA-DR were similar to M1. Additionally, NCI-H460 cells induced a different cell surface marker and cytokine profile than MCF-7 cells.

### VALE and OA modulated the cytokine secretion and cell surface marker expression of tumour cell polarized macrophages

Regarding previously reported immunomodulatory and anti-tumoural properties of pentacyclic triterpenes and the prognostic impact of alternatively polarized TAMs, we were interested in the effect of VALE and OA on the polarization of macrophages. Macrophages were polarized *in vitro* as described above, displaying either specific M2 markers for IL-4/IL-13 polarized macrophages or a mixed profile for NCI-H460 or MCF-7 polarized macrophages. After polarization the macrophages were cultured with either VALE 25 μg/ml or OA 2.5 μg/ml for 3 days.

There was no significant alteration in the cell surface marker profile or the cytokine release of M2 macrophages after treatment with VALE or OA compared to the untreated control.

**Figure 2:**
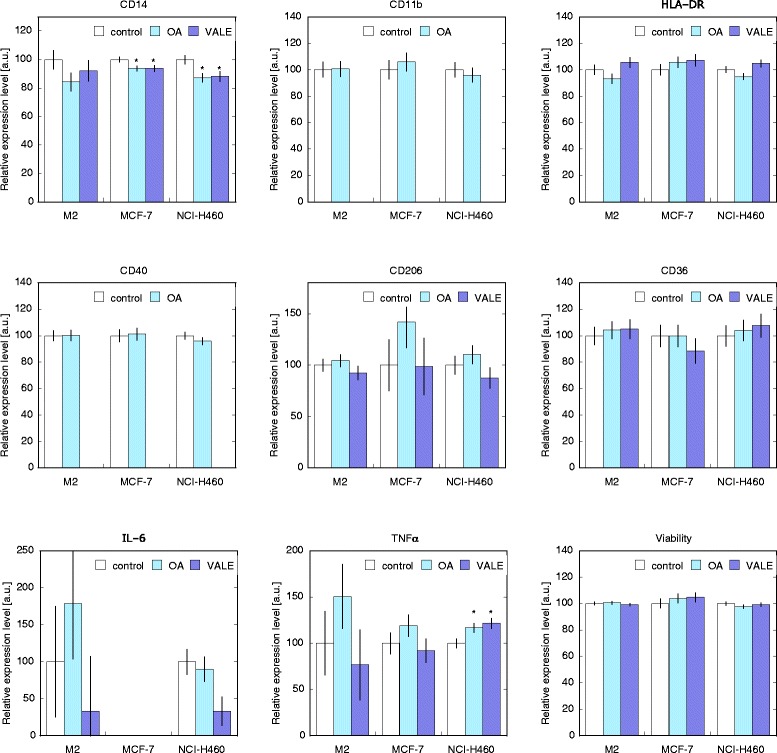
Effect of VALE/OA on cell surface marker expression and cytokine release of polarized macrophages. Macrophages were polarized with cytokines (IL-4/IL-13) for M2 or co-cultured with tumour cells (MCF-7 or NCI-H460) and then cultured with VALE 25 μg/ml or OA 2.5 μg/ml for 3 days before analysis of cell surface markers and cytokines. In cells treated with VALE, CD11b and CD40, measurement, using APC-labelled antibodies, could not be analysed because VALE generated fluorescence artefacts. Mean data of 5 independent experiments represent the expression level relative to the untreated control (mean ± SE, arbitrary units). *p < 0.05 compared to the control (LSD-test).

In MCF-7 polarized macrophages the only alteration by VALE and OA was a modest but significantly lower expression of CD14 (p < 0.05), an effect that was seen in NCI-H460 polarized macrophages as well (p < 0.05). In NCI-H460 polarized macrophages we observed a trend to inhibition of IL-6 release by VALE. Compared to control, TNF-α production was significantly higher in cells treated with VALE and OA (p < 0.05) (Figure 2). Markers measured using APC-labelled antibodies (CD11b and CD40) could not be analysed in cells treated with VALE because VALE generated fluorescence artefacts, identified by isotype controls.

### Supernatants of tumour cell lines influenced monocyte chemotaxis/transmigration

**Figure 3:**
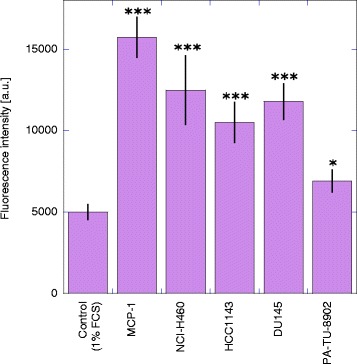
Migration of monocytes induced by tumour cell conditioned media. The effect of conditioned media of human lung carcinoma cell line NCI-H460, breast carcinoma cell line HCC1143, prostate carcinoma cell line DU145 and pancreas adenocarcinoma cell line PA-TU-8902 on monocyte chemotaxis/transmigration in comparison with MCP-1 and medium (1% FCS) alone was measured in a chemotaxis assay. Each bar represents the mean fluorescence intensity ± SD (a.u. = arbitrary units) of 3–5 independent experiments for each cell line. *p < 0.05, ***p < 0.001 compared to the negative control (medium, 1% FCS), (LSD-test).

To study their effect on cell function we investigated the chemotactic potential of several tumour cell conditioned media on the transmigration of monocytes. As chemotactic control we used 25 nM MCP-1. The migratory activity of monocytes in response to MCP-1 was increased about 3-fold compared to spontaneous migration of monocytes in medium alone (p < 0.001). For all cell lines tested monocyte migration was significantly increased compared to medium alone (Figure 3) but was significantly less than monocyte migration due to MCP-1 (p < 0.001). Standardizing the migration to MCP-1 as 100% migratory activity, supernatant from NCI-H460 showed the strongest potential of all investigated cell lines to cause monocyte chemotaxis with 79% chemotactic activity followed by DU145 (75%) and HCC-1143 (67%). PA-TU-8902 cell supernatant showed less chemotactic potential (44%) compared to the other cell lines and the control with 1% FCS (32%).

### VALE/OA inhibited monocyte migration induced by MCP-1 but not by tumour cell conditioned media

**Figure 4:**
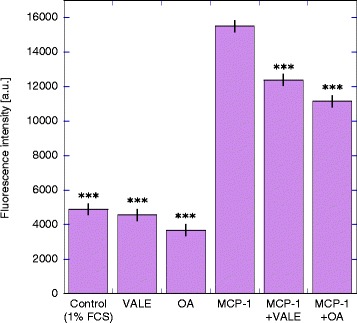
Inhibitory effects of VALE and OA on monocyte transmigration towards MCP-1. The effect of VALE and OA on transmigratory activity and on MCP-1 induced migratory activity of isolated fluorescence labelled monocytes was measured in a chemotaxis assay. Culture medium was used as negative and MCP-1 as positive control. Each bar represents the data of 3–4 independent experiments (mean fluorescence intensity ± SE; a.u. = arbitrary units). ***p < 0.001 compared to MCP-1 (LSD-test).

As shown in Figure 4 VALE or OA alone did not act as chemotactic attractants for monocytes. Migration of monocytes towards medium supplemented with VALE was comparable to the spontaneous migration measured in samples with medium alone. Monocyte transmigration towards OA was significantly lower than the negative control (p < 0.05). Though, monocyte chemotactic protein MCP-1 induced a 3-fold higher transmigratory activity than spontaneously migrating monocytes. The combination of MCP-1 with VALE 50 μg/ml or OA 5 μg/ml led to a significant (p < 0.001) inhibition of monocyte migration (Figure 4). Standardizing the migration data to MCP-1 as 100% migratory activity, the combination of VALE or OA with MCP-1 led to 80 ± 4% (mean ± SE) migratory activity for VALE and to 70 ± 4% migration for OA respectively. Spontaneous migration of monocytes in response to medium (1% FCS) without MCP-1 was 34 ± 4%. OA exhibited a significantly stronger inhibitory effect on monocyte migration compared to VALE (p < 0.05), suggesting OA to be the main component to influence monocyte migration. Concentrations of VALE and OA used in the assay did not affect the viability of monocytes that was >95% in all samples (Table 1), indicating that VALE and OA directly affected the migratory potential of monocytes.

**Table 1:** **Viability of monocytes after treatment with VALE/OA for 2h and 3.5h (mean ± SD)**

**Time**	**Control**	**OA**	**VALE**
2 h	95.0 ± 0.9%	95.1 ± 0.4%	95.7 ± 0.3%
3.5 h	95.9 ± 0.6%	94.7 ± 0.7%	95.7 ± 0.1%

Viability was determined by FACS analysis and results are expressed as percentage of 7-AAD negative cells ± standard deviation.

We also performed the transmigration assay with VALE/OA in combination with tumour cell CMs. Interestingly, neither VALE nor OA had an influence on the migration of monocytes induced by tumour cell supernatants (Table 2).

**Table 2:** **Effect of VALE or OA on monocyte transmigration induced by tumour cell conditioned media**

	**Fluorescence intensity (a.u.)**							
**Monocyte chemotactic transmigration towards:**	**Chemo-attractant (control or TZ-CM)**	**SE**	**Chemo-attractant + VALE**	**SE**	**P-value**	**Chemo-attractant + OA**	**SE**	**P-value**
Neg. control (culture medium)	**6641**	881						
Pos. control (MCP-1)	**16587**	2280						
NCI-H460	**10302**	788	**11399**	953	0.095	**10708**	697	0.531
HCC1143	**9457**	1327	**9925**	1523	0.556	**9843**	2316	0.627
DU145	**11389**	837	**12174**	937	0.229	**11384**	955	0.994
PA-TU-8902	**6743**	894	**6923**	1469	0.820	**7871**	1979	0.160

TZ-CM: tumour cell conditioned medium.

SE: standard error.

a.u.: arbitrary units.

## Discussion

In our *in vitro* study we observed typical M1 or M2 polarization patterns in monocyte derived macrophages generated by IFN-γ/LPS or IL-4/IL-13 and a mixed M1/M2 phenotype after polarization with tumour cells. In transmigration experiments a distinct but individually different chemotactic activity of tumour cell supernatants as well as monocyte chemotactic factor-1 (MCP-1) on monocytes was shown. *Viscum album* lipophilic extract and its predominant triterpene oleanolic acid had minor effects on polarization but significantly inhibited MCP-1 induced monocyte transmigration.

Macrophages display considerable functional plasticity and immediately respond to changes in their microenvironment [5,13]. Based on their pro- or anti-inflammatory abilities, two major types of macrophages, M1 or M2 were described. [36-38]. In the past few years a revision of the strict M1/M2 model was suggested and a continuum of macrophage phenotypes ranging between the two extremes of pro- (M1) and anti-inflammatory (M2) types was proposed [12]. Our results are comparable to other studies on macrophage polarization using cytokines, tumour cells or tumour cell supernatants [11]. CD40 and IL-6, markers characteristic for M1 macrophages, were significantly elevated in IFN-γ/LPS polarized macrophages and the scavenger receptors CD36 and CD206, referred to M2, were significantly higher expressed in IL-4/IL-13 treated macrophages. To further define polarized macrophage patterns we additionally used classical monocyte/macrophage markers. The integrin family member CD11b and the major histocompatibility complex II (MHC II) receptor HLA-DR were significantly increased in M2 compared to M1 macrophages. This finding can be attributed to the influence of IL-4 and IL-13, cytokines that are known to up-regulate the expression of MHC class II receptors and of various adhesion molecules, including CD11b on monocytes and macrophages [39-41].

In our experiments tumour cell co-cultured macrophages displayed both M1 and M2 like characteristics and differed between the two tumour cell lines. Such dual M1/M2 phenotypic patterns have been detected previously for example after i*n vitro* treatment of macrophages differentiated from THP-1 human monocyte cell line and MDM with primary tumour cell culture or tumour cell line supernatants [33] and *in vivo* in histologically normal tissue adjacent to breast tumours [32]. We also found a significant increase in CD14 cell surface expression in MDMs co-cultured with NCI-H460 cells compared to M1, M2 and MCF-7 polarized macrophage populations. Increased expression of cell surface CD14 receptor on local macrophages has been observed in chronic inflammatory conditions [42,43]. This could be the effect of high IL-6 levels that were measured in NCI-H460 but not in MCF-7 conditioned medium (data not shown). IL-6, a cytokine involved in chronic inflammation, was often reported to be present in primary and tumour cell line fluids. Studies on *in vitro* tumour cell induced differentiation of M2 macrophages showed IL-6 dependent differentiation. Furthermore, increased myelomonocytic markers like CD14 and CD163 were detected after macrophage differentiation by soluble mediators of non-small cell lung carcinoma cell lines [44,45].

Although it is well known that homogenous cancer cell lines strongly differ from primary cultures and both do not fully reflect the interaction and regulation mechanisms of *in vivo* tumour microenvironment, such a macrophage phenotype displaying pro- as well as anti-inflammatory features could occur in tumours. Macrophages within tumours are called TAMs (Tumour Associated Macrophages) and have often been associated with an M2 phenotype. However, this may not always properly reflect the real situation as the tumour milieu itself changes during the development of cancer. Depending on the specific tumour microenvironment, TAMs can exert pro-tumorigenic functions such as metastatic seeding, tumour growth and angiogenesis as well as tumour suppressing activity [7,46,47].

Taking the results from *in vitro* investigations as only a part of the more complex *in vivo* situation, it still represents important information about the whole framework of cancers and their microenvironment.

Based on our previous experience with immunomodulatory effects of triterpene containing Viscum album lipophilic extract on monocytes we were interested if it exerted a modulatory potential on macrophage polarization as well. For this purpose we used concentrations of VALE and OA that were established in prior studies on PBMC, monocytes and fibroblasts and proved to be effective but not cytotoxic [48,49]. In our study, CD14 levels were slightly, but significantly decreased after treatment with VALE and OA in both populations of tumour cell co-cultured MDM. The decrease was parallel to diminished (although not significant) IL-6 levels and a significant elevation of TNF-α in NCI-H460 co-cultured MDM. Such an unusual differentially regulated IL-6 down- and TNF-α up-regulation was already described by Tsuboi et al., who observed this phenomenon after treating human PBMCs with nonsteroidal anti-inflammatory drugs (NSAIDs) [50]. In another study increased levels of TNF-α after NSAID treatment were directly attributed to a reduced level of prostaglandin E2 that, although mostly regarded as a pro-inflammatory mediator, also possesses potent anti-inflammatory properties [51]. Further study is necessary to examine, if similar mechanisms could be responsible for the effect of VALE and OA. In general, IL-6 in cancers is associated with proliferation, inhibition of apoptosis, carcinogenesis and conversion of non-cancer cells into tumour stem cells [52,53]. TNF-α is known to have a double-edged role in tumours and is often associated with inducing cancer, angiogenesis, proliferation and metastasis [52-54]. On the other hand high levels of TNF-α are known to act anti-tumoural [52] and it seems as if the pro- or anti-tumour functions of TNF-α are dependent on the site of occurrence in tumours [52,55]. If triterpene containing compounds such as VALE are indeed able to influence cytokine release from tumour cell polarized macrophages, as our in vitro findings suggest, these components should be of interest in search of new therapeutic options targeting TAM polarization.

As tumour cells are known to possess the ability to recruit immune cells, especially monocytes into their microenvironment where they differentiate into TAM´s, we investigated the effect of supernatants from different tumour cell lines on the migration of monocytes in a transwell chemotaxis assay. As positive control for chemotaxis we used MCP-1, that is also referred to as chemokine (C-C motif) ligand 2 (CCL2) and possesses chemotactic activity for monocytes, macrophages and T-lymphocytes. In our study monocytes were attracted by supernatants of tumour cell lines, suggesting soluble factors derived from tumour cells to mediate migration. Our findings are in line with other investigations on the chemotactic potential of tumour cell supernatants on monocytes. Supernatants of colon, breast cancer or myeloid leukemic cell lines were shown to induce significantly different migratory activity of monocytes *in vitro* [6,56]. Supernatants of human ovarian carcinoma cells induced *in vitro* chemotactic activity of monocytes and there seemed to be a correlation between chemotactic activity and tumour-associated macrophage content in ovarian cancer [57]. According to our knowledge, we were first to prove the *in vitro* chemotactic potential for supernatants of the four tumour cell lines we used. As Wang et al. found chemotactic as well as anti-chemotactic regulation mechanisms of supernatants from human sarcoma and ovarian carcinoma cells [58], the differences of migratory activity in the supernatants could be due to diverse factors released by different tumour cell lines.

VALE and OA both significantly decreased the migration of monocytes towards MCP-1 in doses not toxic for these cells. MCP-1 is one of the most prevalent cytokines in tumour microenvironment and has been suggested to be one of the principle determinants of human tumour macrophage content [59], mediating monocyte/macrophage infiltration and thereby promoting tumour progression [60]. Several reviews about chemokines and their relation with cancer suggest targeting MCP-1 related monocyte recruitment as a potential way to suppress tumour growth, TAM infiltration and early relapse [61-64]. The effect of OA was significantly stronger than effect of VALE, suggesting a possibility of triterpenes, especially OA, to influence cell migration and to reduce the attraction of monocytes via MCP-1. The mechanisms by which VALE and OA were able to influence migration are still not understood. Interestingly, Kuonen et al. observed an activation of NIH/3 T3 fibroblasts with VALE and OA, leading to enhanced migratory activity and improved wound closure *in vitro* [49], a finding that may, in regard to the results in our experiments, underline the modulatory activity VALE and OA have on different cell types.

VALE and OA did not have an impact on monocyte migration induced by tumour cell supernatants, giving the assumption that other factors besides MCP-1 influence the attraction of these cells and that the impact of VALE and OA on monocyte migration is somehow correlated with MCP-1 effects. It is already known that soluble factors besides MCP-1 are able to influence monocyte migration. For example, Clahsen et al. showed induction of mouse monocyte migration via IL-6 [65].

Further research on possible mechanisms of VALE- and OA-induced modulation of cell migration could lead to a better understanding of the therapeutic options regarding these triterpenes.

## Conclusion

Our results demonstrated that co-culture with two tumour cell lines might result in a dual macrophage phenotype with M1 as well as M2 patterns, a finding that is important for a better understanding of tumour microenvironment functions. The observed chemotactic potential of tumour cell conditioned media in our study could serve as a model for further research on modulating tumour cell induced monocyte migration. The triterpene containing compound VALE was shown to modulate monocyte chemotactic transmigration and have some immunomodulatory effects on tumour cell co-cultured macrophages *in vitro*, indicating promising possibilities of triterpenes from *Viscum album* L. to contribute in a multimodal concept of anti-cancer therapy in future.
